# Differential Contributions of Empathy to Math Achievement in Women and Men

**DOI:** 10.3389/fpsyg.2019.01941

**Published:** 2019-09-06

**Authors:** Nermine Ghazy, Eleanor Ratner, Miriam Rosenberg-Lee

**Affiliations:** Department of Psychology, Rutgers University, Newark, NJ, United States

**Keywords:** math achievement, STEM – science technology engineering mathematics, gender differences, empathy quotient, systemizing quotient

## Abstract

Mathematics forms a foundation for the science, technology, engineering and mathematics (STEM) fields. While considerable work has identified the individual cognitive and external systemic factors that influence math achievement, less is known about personality-like traits that might contribute to success in mathematics, especially among women. This study examines two such traits: systemizing – the tendency to analyze systems and extract underlying rules that govern their behavior – and empathizing – the ability to identify with another’s emotions and respond appropriately. Recently Escovar et al. (2016) found that empathizing was a negative predictor of math skills in children, especially among girls, suggesting that women with higher empathy might be particularly disposed to lower math performance. In the first study, 142 participants (71 female) completed two standardized measures of math achievement and questionnaires to gauge the tendency to empathize and systemize. Surprisingly, higher empathy was associated with better math performance in women, while men displayed the expected pattern of lower empathy being related to higher math scores. In a second study, we extend this finding in women (*n* = 121) to show that individuals who report higher mathematics achievement in university level course work also have higher empathy scores. Further, while positive attitudes toward mathematics tended to decline from elementary school to college, women whose attitudes increased had higher empathy scores than those who declined. Together, these results suggest that while the tendency to empathize is associated with worse math performance in childhood, it may become a protective factor as women progress through their mathematics education.

## Introduction

At the undergraduate level, fewer women choose science, technology, engineering, and mathematics (STEM) majors and among those who do pursue these fields, many are diverted toward teaching over higher status STEM careers, such as computer science and engineering (Beede et al., 2011). Moreover, women who do work in STEM are more likely to have lower wages than their male counterparts (Beede et al., 2011). At the elementary school level, boys and girls perform equivalently, but the genders diverge over the course of schooling (Hyde et al., 1990), and ultimately, mathematics has among the lowest female participation rate at the graduate level (Leslie et al., 2015).

Considerable research to date has focused on factors that contribute to low STEM participation rates in the United States, such as a lack of female role models (Cheryan et al., 2011) and how gender stereotypes may subconsciously discourage women from pursuing STEM fields (Shapiro and Williams, 2012). These important external, systemic factors interact with internal, individual factors to impact academic achievement and eventually predispose some individuals to avoid STEM careers. Mathematics achievement is a strong predictor of STEM career attainment (Crisp et al., 2009), making it vital to understand how individual differences in personality-like traits influence math achievement and hence pathways to STEM careers.

This study examines two such traits: *systemizing* – the tendency to analyze systems and extract underlying rules that govern their behavior – and *empathizing* – the ability to identify with another’s emotions and respond appropriately. While systemizing is hypothesized to relate to math skills (Baron-Cohen, 2009), Escovar et al. (2016) recently found no evidence for a direct relationship in a large sample of children. Instead, they reported that higher levels of empathizing were related to lower math skills, especially in girls. The authors hypothesized that girls who were more attuned to the social environment may be particularly susceptible to stereotyped messages about gender and mathematics, potentially impacting performance over the course of schooling and ultimately lowering participation in STEM fields. Thus, the primary goal of the current study was to examine whether a negative relationship between empathizing and math achievement held in female college students.

### The Role of Empathizing and Systemizing in Math Cognition

Theorizing on the role of systemizing and empathizing in math cognition grows out of research on autism spectrum disorder (ASD), a neurodevelopmental disorder marked by social communication deficits and repetitive behaviors or unusual interests (American Psychiatric Association, 2013). Notably, individuals with autism often have remarkable cognitive strengths in domains such as memory, spatial reasoning and mathematics (Treffert, 2009; Iuculano et al., 2014). Baron-Cohen (2009) has suggested that this constellation of behaviors can be related to difficulties empathizing with others, but a relative strength in systematic thinking. Mathematics, with its emphasis on rules and structures is a paradigmatic example of a system, suggesting that individual differences in systemizing should be positively related to variation in mathematics skills. While several studies find increases in systemizing among individuals with autism (Wakabayashi et al., 2007; Auyeung et al., 2009; Greenberg et al., 2018) and higher rates of autistic-like behaviors in mathematicians (Baron-Cohen et al., 2001), there is surprisingly little empirical support relating systemizing directly to math skills among either typically developing individuals or those on the autism spectrum. In fact, the one study to examine this question directly in adult females found no correlation between the tendency to systemize and math achievement (Morsanyi et al., 2012).

In children, Escovar et al. (2016) did find a marginally significant positive relationship between parents’ reports on the Systemizing Quotient (SQ) and a measure of mathematical reasoning. However, this weak relation was entirely accounted for by shared variance with measures of intelligence and reading ability. The child version of the SQ is combined with the Empathy Quotient (EQ) into a single instrument, so Escovar et al. (2016) also assessed the relationship between participants’ tendencies to empathize and math achievement. Contrary to the prediction of no relationship, they reported that greater empathizing was related to lower performance on a composite of math calculation skills (comprising both timed and untimed measures), and this relation remained even after accounting for intelligence and reading ability (Escovar et al., 2016). Consistent with prior research (Lawrence et al., 2004; Auyeung et al., 2009), girls had significantly higher scores on the EQ, moreover, the relationship between EQ and math scores was stronger in girls than boys, although this interaction effect did not reach significance.

### Explaining the Influence of Empathizing on Math Achievement

To further explore the relationships between gender, EQ, and math scores, Escovar et al. (2016) considered the role of math anxiety, a robust predictor of math achievement (Ashcraft, 2002; Wu et al., 2012). Potentially, individuals who are more empathetically tuned may be more prone to experience math anxiety, thus explaining the relationship between EQ and math achievement. Consistent with prior research (Ma, 1999; Wu et al., 2012), math anxiety predicted math performance, but this relationship was independent of the influence of empathizing (Escovar et al., 2016). Instead, they found that parents’ report of children’s social skills were strongly related to empathizing and mediated the relationship between empathizing and math skills, an effect that was significantly stronger in girls than boys.

Empathy is not a unitary concept and modern approaches define both cognitive empathy, the ability adopt another’s perspective and recognize and label their mental state, and emotional empathy (also termed affective empathy or emotional reactivity), the tendency produce the appropriate emotional response to another’s emotional state (Rueda et al., 2015). A third category, social skills, describes using the appropriate behaviors in response to the emotions of others (Lawrence et al., 2004). Using factor analysis, Lawrence et al. (2004) established that these three components can be assessed from subsets of the EQ items. In the current study, we sought to further explore the relationship between empathy and math achievement by considering its relationship to these three components.^1^ More broadly, a second goal of this study was to understand the factors contributing to the relationship between math achievement and empathy, with respect to math anxiety, social skills and distinct components of empathizing.

### Gender Gap in Math Achievement

The gender gap in mathematics achievement is not fixed over the course of schooling. In elementary school there are no consistent differences between the genders (Bakker et al., 2019), but the gap grows in middle school, high school and university (Hyde et al., 1990), with men outperforming women. One potential explanation for this pattern is that social, rather than cognitive factors, grow in their influence on women’s math achievement.

The phenomenon of stereotype threat – in which awareness of gender stereotypes can reduce performance in stereotyped groups – suggests that individuals with stronger tendencies to be aware of the feelings of others (as in the EQ) and cognizant of social expectations (a component of social skills) may be particularly prone to internalizing these stereotypes by the time they reach adulthood. For example, Beilock et al. (2010) found that students of teachers with greater math anxiety had smaller gains in math achievement over the school year, but only among students who endorsed gender stereotypes. Attitudes toward mathematics are an individual difference trait that also correlates with current math achievement and future math performance (Anttonen, 1969; Ma and Kishor, 1997). We reasoned that among women, changing attitudes toward mathematics may be a rough index of internalization of societal messages about gender and mathematics. Thus, a third goal of this study was to assess whether changes in women’s attitudes toward mathematics over the course of schooling impact the relationship between empathizing and math achievement in adulthood.

### The Current Studies

In this paper, we sought to investigate the relationship between math achievement and empathizing, systemizing and social skills in adults. In Study 1, we tested whether the association of higher empathy with lower math skills found in children also holds in young adults, especially women. In Study 2, we sought to replicate the results of Study 1 and examine the role of changing attitudes toward mathematics on the relationship between empathizing and math achievement in women.

## Study 1

### Methods and Procedures

#### Participants

One Hundred and forty two college-aged students (71 women) attending Rutgers University in Newark, NJ, United States, ranging in age from 18 to 25, participated in this study. Participants received credit toward their psychology coursework.

#### Standardized Mathematics Assessments

Math skills were measured using two subtests of the Woodcock Johnson – 3rd Edition (WJ-III): Calculation and Math Fluency. While both measures were collected by Escovar et al. (2016), that study combined them into a single calculation composite score. Here, we analyzed the measures separately, in order to assess differential effects on timed and untimed math skills. Calculation, a non-timed test, measures a person’s ability to complete mathematical problems that increase in difficulty starting from basic arithmetic up to calculus. Math Fluency, a 3-min timed test of single-digit arithmetic problems, requires answering intermixed addition, subtraction, and multiplication questions. Raw scores were converted to age based standardized scores using Woodcock Johnson Compuscore software.

#### Empathizing Measure

The EQ (Baron-Cohen and Wheelwright, 2004) is a 40-item questionnaire for adults designed to measure a person’s empathy. Empathy is defined as being able to identify with another person’s emotions and perspective. Responses are given using a 4-point Likert scale (*definitely agree, slightly agree, slightly disagree*, and *definitely disagree*), and a subset of items are reverse coded to reduce acquiescence bias. Participants receive a “0” for a response that does not endorse the trait, and a “1” or “2” for a response that endorses the trait, depending on the strength of the reply. Missing items were replaced by the participants’ average score on all other items in that measure. For the EQ, there were ten instances of items missing (0.18% of items) and no participants missed more than one item. Internal reliability was good (Cronbach’s α = 0.831).

To investigate distinct components of empathy, namely cognitive empathy (EQ-CE), emotional empathy (EQ-EE) and social skills (EQ-SS), we summed scores on the items identified by the maximum factor loadings in Lawrence et al. (2004). This division of items corresponds to the approach taken in a large study of the psychometric properties of the EQ in a Dutch sample (Groen et al., 2015). Further, assigning items uniquely to components reduces the correlation between the components, allowing us to better assess their independent contributions to math skills. Items in the EQ-CE component probe understanding of the mental states of others (*I can pick up quickly if someone says one thing but means another*), while EQ-EE items assess appropriate emotional reactions (*I get upset if I see people suffering on news programs*). Finally, items on the EQ-SS index ease in social situations (*I don’t tend to find social situations confusing*).

#### Systemizing Measure

The SQ (Baron-Cohen et al., 2003) is a 75-item adult self-report questionnaire designed to measure a person’s ability to systemize. Systemizing is defined as the tendency to analyze or construct organized schemes. Responses are given using the same 4-point Likert scale as the EQ and a subset of items are reverse coded to reduce acquiescence bias. For the SQ, there were 26 instances of items missing (0.24%) and one participant was missing four items, six were missing two items and the remaining participants missed one item. Scoring follows the same procedures as for the EQ and missing items were replaced by the participants’ average score on all other items in the measure. Internal reliability for the SQ was good (Cronbach’s α = 0.892).

#### Math Anxiety Measure

The Abbreviated Math Anxiety Research Scale (A-MARS, Alexander and Martray, 1989) is a 25-item questionnaire for adults measuring math anxiety. Participants are asked to rate their level of anxiety in a variety of situations, such as studying for and taking math tests, using a 5-point Likert scale (*not at all, a little, a fair amount, much*, and *very much*). Total score is the sum of responses and missing values were replaced by that participant’s average on other items. There were six instances of items missing (0.17%) and no participants were missing more than one item. Cronbach’s alpha for the A-MARS was 0.957.

#### Social Abilities Measure

The adult self-report version of the social responsiveness scale (SRS, Constantino and Gruber, 2012) was used to broadly assess social skills. Each question on this 65-item measure describes a social behavior or characteristic and uses a 0- to 3-point Likert scale (*not true, sometimes true, often true*, and *almost always true*). Higher scores on the SRS correspond to worse social skills, thus items describing prosocial behaviors were reverse scored. We employed the total raw scores for subsequent analyses, and missing values were replaced by that participant’s average on all other items. There were 10 instances of items missing (0.22%), one participant was missing three items, two were missing two items, and the remaining participants missed one item. Cronbach’s alpha for the SRS was 0.908.

#### Demographics Questionnaire

Participants were asked to report their assigned sex status at birth (Male, Female, Intersex) and their gender identification (Male, Female, Gender Variant). No participants reported being assigned intersex at birth, nor identified as gender variant, and all participants reported assigned sex and gender identification matched and were used in subsequent analyses. Participants were also asked about race and social-economic status.

#### Regression Analyses

Multiple regression analyses were performed to assess the interaction of gender with EQ and SQ in predicting math skills. We first modeled the main effects of gender, EQ and SQ on the Calculation and Math Fluency math measures. Next, we added terms for the interaction of Gender with EQ and Gender with SQ to the model, using dummy coding to indicate female participants. To explore the effects of math anxiety and SRS on Calculation, we also computed main effect models including those terms. And then asked whether adding the interaction terms explained additional variance. To examine the independent contributions of the EQ components on Calculation and Math Fluency, we first modeled the main effects of all the components, along with gender and then added all the interaction terms to the model. Covariates were mean centered before being entered into the model, reducing multicollinearity between predictors, as indicated by variance inflation factors less than 4 in all models (Wood, 1984).

#### Procedure

Participants completed the Math Fluency and Calculation subtests of the WJ-III, using pencil and paper. Participants then used a laptop computer to complete a series of questionnaires implemented in Qualtrics (Provo, UT, United States). Participants completed the EQ, SQ, A-MARS, SRS, and the basic demographics questionnaire. The full session took 50–60 min.

### Results

#### Gender Differences in Math Achievement, Systemizing and Empathizing

Participant characteristics are listed Table 1. Women scored significantly lower than men in the untimed Calculation and in timed Math Fluency subtests of the WJ-III). Women scored significantly lower on the SQ than men. While women had numerically higher scores on the EQ, this difference did not reach significance (*p* = 0.108).

**TABLE 1:** Participant characteristics (Study 1).

	**Women (*n* = 71)**		**Men (*n* = 71)**		*****t*****	***p***
	***M***	***SD***	***M***	***SD***		
Age^1^	20.370	1.819	20.280	1.870	–0.287	0.774
Calculation	104.324	12.334	108.464	11.846	2.040	0.043
Math Fluency	94.930	13.364	101.690	13.593	2.988	0.003
EQ	45.004	10.583	42.195	10.134	–1.616	0.108
SQ	59.932	18.904	67.443	18.968	2.363	0.019
Math Anxiety	64.952	19.580	60.672	21.911	–1.227	0.222
SRS	111.325	21.259	116.463	23.510	1.366	0.174

*EQ, Empathy Quotient; SQ, Systemizing Quotient; SRS, Social Responsiveness Scale; ^1^Two female participants did not report their date of birth; therefore, the sample size for women is *n* = 69.*

#### Relations Between Math Achievement, Systemizing and Empathizing

Table 2 lists the correlations between SQ, EQ and math performances in the full sample. Scores on the EQ and SQ were positively correlated, but there was no relationship between EQ or SQ and either of the math measures (all *p*s > 0.13).

**TABLE 2:** Pearson correlations between math achievement, EQ, SQ, Math Anxiety, and SRS (Study 1).

	**Math Fluency**	**EQ**	**SQ**	**Math Anxiety**	**SRS**
**Full Sample**					
Calculation	0.464^∗∗^	–0.027	–0.028	−0.214^∗^	0.078
Math Fluency		0.118	0.126	–0.284^∗∗^	–0.111
EQ			0.332^∗∗^	−0.193^∗^	–0.580^∗∗∗^
SQ				−0.203^∗^	–0.147
Math Anxiety					0.366^∗∗^
**Women**					
Calculation	0.534^∗∗^	0.233	0.058	−0.282^∗^	–0.189
Math Fluency		0.313^∗∗^	0.273^∗^	–0.226	–0.336^∗∗^
EQ			0.300^∗^	–0.119	–0.600^∗∗∗^
SQ				–0.362^∗∗^	−0.264^∗^
Math Anxiety					0.312^∗∗^
**Men**					
Calculation	0.349^∗∗^	−0.262^∗^	–0.190	–0.126	0.294^∗^
Math Fluency		–0.002	–0.104	–0.306^∗∗^	0.026
EQ			0.442^∗∗∗^	−0.297^∗^	–0.551^∗∗∗^
SQ				–0.033	–0.093
Math Anxiety					0.439^∗∗∗^

*Top panel: Full sample. Middle panel: Women. Bottom panel: Men. Abbreviations as in Table 1. ^∗^Correlation is significant at the 0.05 level (two-tailed). ^∗∗^Correlation is significant at the 0.01 level (two-tailed). ^∗∗∗^Correlation is significant at the 0.001 level (two-tailed).*

#### Gender Differences in Relations Between Empathizing, Systemizing and Math Achievement

**FIGURE 1:**
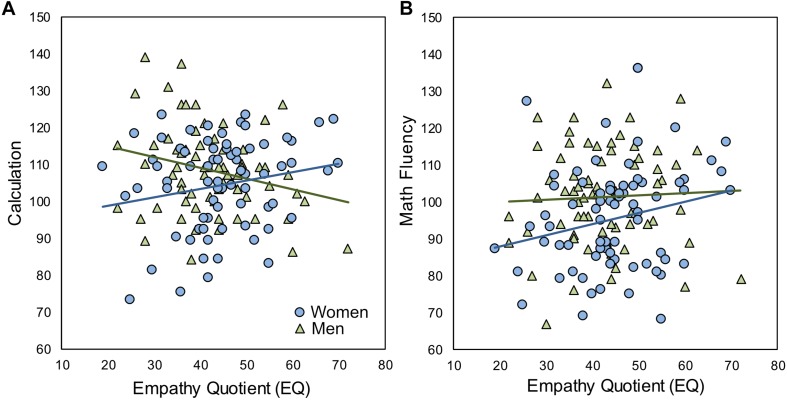
Relations between EQ and Math Achievement (Study 1). **(A)** Women had a positive relationship between performance on the Calculation subtest of the WJ-III and empathy quotient [*r*(71) = 0.233, *p* = 0.051], while men displayed the opposite pattern [*r*(71) = –0.262, *p* = 0.028]. **(B)** Women had a positive relationship between performance on the Math Fluency subtest of the WJ-III and empathy quotient [*r*(71) = 0.313, *p* = 0.008], while there was no relationship in men [*r*(71) = −0.0020, *p* 0.984].

Next, we considered the relations between EQ, SQ and math achievement, separately in women and men. Here, we found divergent results between the genders, with positive relations of EQ and SQ with math achievement in women, and negative relationships in men (Table 2 and Figure 1). Most notably, in women there was a strong positive relationship between EQ and Math Fluency and a marginal relationship with Calculation. While in men, the opposite pattern held, namely lower empathizing was related to higher math achievement for Calculation, although there was no relationship for Math Fluency. For systemizing, we found the same pattern of positive correlations in women and negative correlations in men, but only the relationship between SQ and Math Fluency in women was statistically significant (Table 2).

To confirm this pattern of results, we employed multiple regression analyses to determine whether EQ, SQ, or both, interacted with gender in predicting participants’ math scores. For Calculation, in Model 1, there was a main effect of gender and as expected no significant effects for EQ or SQ. Adding the interaction terms produced a significant F-change [*F*(2,136) = 4.36, *p* = 0.015] and there was a significant interaction between gender and EQ, but no interaction between gender and SQ (Table 3, Model 2). A follow-up analysis, using only EQ, gender and their interaction in predicting Calculation, revealed a main significant negative effect of EQ in men (*t* = −2.20, *p* = 0.030) and a significant positive effect in women (*t* = 3.00, *p* = 0.003), as well as a significant effect of gender (*t* = −2.05, *p* = 0.043). For Math Fluency, there was again a main effect of gender, but no effects of EQ or SQ (Supplementary Table S1). Adding the interaction terms produced a significant F-change [*F*(2,136) = 3.43, *p* = 0.035], but only the interaction of gender and SQ was marginally significant.

**TABLE 3:** Hierarchical regression analysis of Calculation.

	***B***	***SE***	**B eta**	***t***	***p***
**Model 1 R^2^ = 0.033**					
Intercept	108.68	1.48		73.69	< 0.001
Gender	–4.56	2.13	–0.18	–2.14	0.034
EQ	0.03	0.107	0.02	0.49	0.805
SQ	–0.05	0.06	–0.07	–0.79	0.432
**Model 2 R^2^ = 0.091, Sig (F-change_model__1_) = 0.015**					
Intercept	108.32	1.48		73.19	< 0.001
Gender	–4.42	2.08	–0.18	–2.12	0.036
EQ	–0.26	0.16	–0.22	–1.65	0.101
SQ	–0.06	–0.08	–0.09	–0.69	0.489
Gender ^∗^ EQ	0.53	0.210	0.33	2.54	0.012
Gender ^∗^ SQ	0.05	0.12	0.05	0.43	0.668

*Model 1: Effects of gender, empathy quotient (EQ) and systemizing quotient (SQ). Model 2: additional variance accounted for by interaction of Gender with SQ and EQ.*

#### Gender Differences in Relations Between Empathizing and Math Achievement Remain After Controlling for Math Anxiety

Next, we examined the role of math anxiety on the relationship between EQ and Calculation. Consistent with prior research, we found a negative correlation between scores on the A-MARS and performance on Calculation and Math Fluency, in the full sample (Table 2). We also found that math anxiety was negatively related to EQ and SQ (Table 2). Thus, we sought to determine if math anxiety might explain the relationship between gender and EQ in predicted Calculation scores. Adding math anxiety to the multiple regression model predicting Calculation (Model 3) revealed a main effect of math anxiety and a significant F-change relative to Model 1 [*F*(1,137) = 6.70, *p* = 0.011]. However, adding the interaction terms to this model still resulted in a significant F-change [*F*(2,135) = 5.41, *p* =0.005] and the interaction between EQ and gender remained significant (Table 4, Model 4). Notably, there was also a main effect of EQ in this model, indicating a significant negative relationship between EQ and Calculation in men, as well as the positive relationship in women denoted by the interaction term. These results suggest the role of empathizing on math achievement is not explained by math anxiety.

**TABLE 4:** Hierarchical regression analysis of Calculation accounting for Math Anxiety.

	***B***	***SE***	**Beta**	***t***	***p***
**Model 3 R^2^ = 0.078 Sig (F-change_model1_) = 0.011**					
Intercept	108.41	1.45		74.818	< 0.001
Gender	–4.03	2.10	–0.17	–1.92	0.057
EQ	–0.02	0.11	–0.01	–0.15	0.881
SQ	–0.06	0.06	−0.10	–1.11	0.268
Math Anxiety	–0.13	0.05	–0.22	–2.59	0.011
**Model 4 R^2^ = 0.147, Sig (F-change_model3_) = 0.005**					
Intercept	107.77	1.45		74.24	< 0.001
Gender	–3.75	2.04	–0.15	–1.84	0.068
EQ	–0.37	0.16	–0.12	–2.37	0.019
SQ	–0.04	0.08	–0.06	–0.45	0.651
Math Anxiety	–0.15	0.05	–0.25	–2.96	0.004
Gender ^∗^ EQ	0.64	0.21	0.36	3.10	0.002
Gender ^∗^ SQ	–0.03	0.11	–0.03	–0.23	0.818

*Calculation Model 3: Effects of gender, empathy quotient (EQ), systemizing quotient (SQ) and math anxiety. Model 4: additional variance accounted for by interaction of Gender with SQ and EQ.*

#### Gender Differences in Relations Between Empathizing and Math Achievement Are Explained by Social Skills

Next, we examined the role of social skills, as measured by the SRS, on the relationship between EQ and Calculation. Higher scores on the SRS indicate worse social skills and EQ was negatively correlated with SRS in the full group (Table 2) and in each gender (Table 2). A model (Table 5, Model 5) which included a main effect of SRS scores did not significantly improve the fit relative to Model 1 [*F*(1,137) = 0.75, *p* =0.390], however adding the interaction of SRS and gender to that model (Table 5, Model 6) resulted in significant F-change [*F*(1,136) = 8.47, *p* = 0.004] in the predictions of Calculation and a significant interaction between gender and SRS. A follow-up analysis, using only SRS, gender and their interaction in predicting Calculation, revealed a significant positive effect of SRS in men (*t* = 2.46, *p* = 0.015) and a significant negative effect in women (*t* = −2.88, *p* = 0.005), as well as a significant effect of gender (*t* = −2.03, *p* = 0.045). As higher SRS scores indicate worse social skills, these results show, consistent with the EQ, that worse social skills are related to better math performance in men, but the opposite pattern in women.

**TABLE 5:** Hierarchical regression analysis of Calculation accounting for SRS.

	***B***	***SE***	**Beta**	***t***	***p***
**Model 5 R^2^ = 0.038 Sig (F-change_model__1_) = 0.390**					
Intercept	108.65	1.48		76.59	< 0.001
Gender	–4.50	2.13	–0.19	–2.11	0.037
EQ	0.09	0.13	0.08	0.69	0.494
SQ	–0.05	0.06	–0.08	–0.83	0.408
SRS	0.05	0.06	0.09	0.86	0.390
**Model 6 R^2^ = 0.095, Sig (F-change_model5_) = 0.004**					
Intercept	108.38	1.44		75.22	< 0.001
Gender	–4.65	2.08	–0.19	–2.24	0.027
EQ	0.07	0.13	0.06	0.55	0.580
SQ	–0.06	0.06	–0.10	–1.06	0.290
SRS	0.16	0.07	0.29	2.40	0.018
Gender ^∗^ SRS	–0.26	0.09	–0.32	–2.91	0.004
**Model 7 R^2^ = 0.112, Sig (F-change_model6_) = 0.276**					
Intercept	108.37	1.47		73.51	< 0.001
Gender	–4.52	2.08	–0.19	–2.18	0.031
EQ	–0.08	0.19	–0.07	–0.41	0.685
SQ	–0.09	0.09	–0.14	–1.02	0.310
SRS	0.12	0.07	0.23	1.68	0.096
Gender ^∗^ SRS	–0.17	0.11	–0.21	–1.52	0.131
Gender ^∗^ EQ	0.30	0.25	0.18	1.18	0.240
Gender ^∗^ SQ	0.07	0.12	0.08	0.63	0.529

*Model 5: Effects of gender, empathy quotient (EQ), systemizing quotient (SQ) and social responsiveness scale (SRS) scores. Model 6: additional variance accounted for by interaction of Gender with SRS. Model 7: additional variance accounted for by interaction of Gender with SQ and EQ.*

Finally, we added terms for the interaction of gender with EQ and SQ to Model 6, which did not produce a significant F-change [*F*(2,134) = 1.30, *p* = 0.279], and none of the gender interaction terms were significant (Table 5, Model 7). Together, these results suggest that both SRS and EQ interact with gender in predicting math skills; however, because of the multicollinearity between the measures, both the main effects and interactions are not significant when included in the same model.

#### Gender Differences in the Relations Between Emotional Empathy and Math Achievement

**FIGURE 2:**
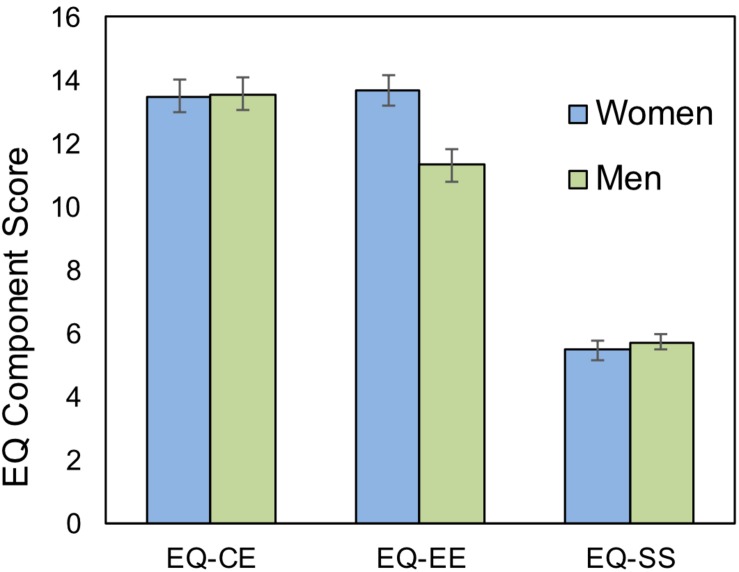
Scores on the Empathy Quotient (EQ) component scores (Study 1). Women scored higher than men on the Emotional Empathy (EE) component of the EQ, but there was no differences on the Cognitive Empathy (CE) and Social Skills (SS) components.

To assess the construct validity of the EQ components of Cognitive Empathy (EQ-CE), Emotional Empathy (EQ-EE) and Social Skills (EQ-SS), we first examined their correlation with SRS scores. As SRS is an index of social skills, we expected the strongest relationship to be between SRS and the EQ-SS component. Indeed, we found that SRS correlated most strongly with EQ-SS [*r*(142) = −0.532, *p* < 0.001], then with EQ-EE [*r*(142) = −0.423, *p* < 0.001] and finally with EQ-CE [*r*(142) = −0.313, *p* < 0.001]. Next we examined gender differences between the EQ components. While there was no significant difference between men and women on the full EQ (Table 1), women had significantly higher scores on the EQ-EE component [*t*(140) = −3.557, *p* = 0.001], but not EQ-CE or EQ-SS (all *p*s > 0.53, see Figure 2).

In unplanned follow-up analyses, we sought to assess which aspect of the EQ drove the positive relationship between empathy and mathematical achievement, in women. In the full sample, there was a marginal positive relationship between EE-SS and Math Fluency [*r*(142) = 0.152, *p* = 0.071]. None of the other components were related to math achievement in the full sample (all *p*s > 0.28). However, in women, the EQ-EE correlated significantly with math achievement for both Calculation and Math Fluency and there was a marginal relationship between EQ-SS and Math Fluency (Supplementary Table S2). In men there was a marginal negative relationship between EQ-SS and Calculation (Supplementary Table S2). No other components correlated with math achievement in men.

To further quantify these gender differences, we performed multiple regression analyses using the three EQ components with the two math measures as dependent variables. In a first model we examined the main effects of gender and each of the components; we then added the interaction terms of each of the components to the model. For Calculation, adding the interaction terms resulted in a significant F-change relative to Model S1 [*F*(3,134) = 4.26, *p* = 0.007, Supplementary Table S3, Model S2] and there was a significant interaction of gender and the EQ-EE component, and marginal interactions of gender with EQ-CE and EQ-SS. For Math Fluency, adding the interaction terms resulted in a marginal F-change relative to Model S3 [*F*(3,134) = 2.58, *p* = 0.056] and only EQ-EE interacted significantly with gender (Supplementary Table S4, Model S4).

### Discussion

In Study 1, we examined the relationship between empathizing, systemizing and math achievement in women and men. We found that among women, both EQ and SQ were positively related to math achievement while the opposite pattern held in men. There was a significant interaction between gender and empathizing in the prediction of Calculation scores, but not Math Fluency, and a marginal interaction between gender and SQ in predicting Math Fluency, but no effect in Calculation. Moreover, the results for Calculation were independent of the strong negative relationship between math anxiety and math achievement found in both men and women.

These results in women are surprising given the negative relationship between math achievement and empathizing found in children by Escovar et al. (2016). Although there was no significant difference in the strength of these effects between boys and girls, follow up analyses found a stronger role for social skills among girls than boys on math performance. Specifically, social skills mediated the relationship between EQ and math achievement in girls, but not boys. Those results are in line with a negative relationship of math achievement with empathizing and social skills more broadly in females. Yet, in the current study we found a positive relationship in women. Moreover, SRS also tracked with EQ in this sample, that is, better social skills were related to better math performance in women, and worse performance in men. Further, including SRS and EQ in the same model (Model 7) removed the interaction between EQ and gender because of the multicollinearity between EQ and SRS.

Additional support for the role of social skills in math performance came from the examination of EQ components scores. There was a marginal relationship between EQ-SS scores and Math Fluency in the full sample and a marginal interaction between gender and EQ-SS in Calculation scores. Less expected were the gender differences in EQ-EE, both in total scores and in the interaction between gender and EQ-EE in predicting Math Fluency and Calculation scores. Cognitive empathy has been associated with theory of mind (Lawrence et al., 2004), which has been linked to executive functions (Perner and Lang, 1999), and we found a marginal interaction between gender and this component in predicting Calculation scores.

Given the unexpected positive relationship between empathizing and math skills found among women in Study 1, we conducted a second study aimed at replicating the direction of this effect. We also sought to assess the role of another individual difference trait known to be related to math skills: attitudes toward mathematics. Surveys of attitudes toward math assess the thoughts and feelings that students bring to mathematics beyond whether it induces anxiety. Notably, attitudes toward math predict math achievement both concurrently (Ma and Kishor, 1997) and longitudinally (Anttonen, 1969) and tend to decline over the course of schooling (Watt, 2004). Further, females tend to have lower levels of positive attitudes toward math (Watt, 2004). Coupled with the growing gap in achievement between the genders starting in middle school (Hyde et al., 1990), these findings suggest that changes in attitudes toward mathematics over the course of schooling may correspond to the internalization of gender stereotypes regarding math achievement. Thus, we asked participants to retrospect over their attitudes from elementary school to the college level.

Our primary goal in Study 2 to was to replicate the unexpected finding of a positive relationship between EQ and math achievement in female college students. Therefore, we restricted our sample to women only. Further, our online method of data collection precluded obtaining standardized measures of math achievement. Instead, we asked students to report their grade point averages (GPA) on college-level mathematics coursework, as well as complete questionnaire measures. Our second goal, was to replicate the association between the emotional empathy components of the EQ and math achievement. Our final goal was to examine the relationship between changes in attitudes toward mathematics and math skills and see if that could explain the relationship between EQ and math achievement in women. We predicted that positive attitudes would decline from elementary school into adulthood, as this is a crucial period when women may be discouraged from pursuing STEM careers. Further, we explored whether changing attitudes toward mathematics could explain the positive relationship between empathizing and math achievement found in Study 1.

## Study 2

### Methods and Procedures

#### Participants

Hundred and twenty one college-aged female students attending Rutgers-University in Newark, NJ, United States, participated in the study, ranging in age from 18 and 27. As in Study 1, participants received credit toward their psychology coursework.

#### Empathizing, Systemizing and Math Anxiety Measures

Scoring and missing data procedures for the EQ, SQ, and A-MARS followed the same procedures as in Study 1, including computing the component scores from the EQ. Six participants were missing one item (0.12%) and Cronbach’s alpha for EQ was 0.845. For the SQ there were 21 instances of missing items, two participants were missing two items and the remainder were missing one for a total of 0.23% missing data. Cronbach’s alpha for the SQ was 0.832. For the A-MARS, 7 participants were missing one item for a total of 0.24% missing data and Cronbach’s alpha was 0.964.

#### Retrospective Attitude Toward Math Measure

The Attitudes Toward Mathematics questionnaire is a 40-item instrument that measures a person’s attitudes toward mathematics beyond math anxiety (Tapia and Marsh, 2004). We adapted this instrument to produce a brief measure of participants’ attitudes toward math over the course of schooling, which we termed Retrospective Attitudes Toward Mathematics (R-ATM). Participants were asked to rate statements on 5-point Likert scale (*strongly disagree, disagree, neutral, agree*, and *strongly agree*) regarding three core attitudes toward math: ability (*I was good at mathematics*), enjoyment (*I liked mathematics*) and importance (*I thought it was important to do well in math class*). We asked participants to retrospect over four stages of schooling: elementary school, middle school, high school and college. Higher values reflect more positive attitudes toward math. We computed a measure of participants change in attitudes by fitting a line to the four time points, using the formula for slope in a linear regression. There were five instances of missing items (0.41%), one participant was missing all three items from elementary school and two other participants were missing one item each. Missing items were replaced by the average of the other items for that time bin. The participant missing all elementary school items was excluded from the repeated measures ANOVA, but included in the slope analyses. The Cronbach’s alpha for the R-ATM was 0.882.

#### Math Measure

Participants were asked to report their GPA on college level math courses. Previous research has found high correlations between students self-report academic grades and their actual grades, with slightly higher reliability for mathematics relative to language studies (Sticca et al., 2017). We only considered responses from participants who reported results on the 4.0 scale used at Rutgers University – Newark (*n* = 80). Math GPA deviated from normality (*skewness* = −0.774, *SE* = 0.269; *kurtosis* = 0.500, *SE* = 0.532), so following Coyle et al. (2011) we computed the square of Math GPA (*skewness* = −0.162, *SE* = 0.269; *kurtosis* = −0.755, *SE* = 0.532). As deviations from normality can distort error estimates we used the transformed (squared) Math GPAs for the analyses, but report the original values in Table 6.

**TABLE 6:** Participant characteristics (Study 2).

	***M***	***SD***
Age^1^	20.864	1.927
Math GPA^2^	3.103	0.712
EQ	44.975	10.401
SQ	57.924	15.104
Math Anxiety	71.625	24.062

*GPA, Grade Point Average. Abbreviations as in Table 1. ^1^Three participants did not report their date of birth; therefore, the sample size is *n* = 118. ^2^Only 80 participants reported Math GPA on 4.0 scale.*

#### Procedure

Data were collected online and consisted entirely of questionnaires and demographic information. Participants completed the EQ, SQ, and R-ATM. Finally, participants completed the demographics questionnaire, including their GPA in college level math. The procedure took 30–45 min.

### Results

#### Relations Between Math Achievement, Systemizing and Empathizing

**FIGURE 3:**
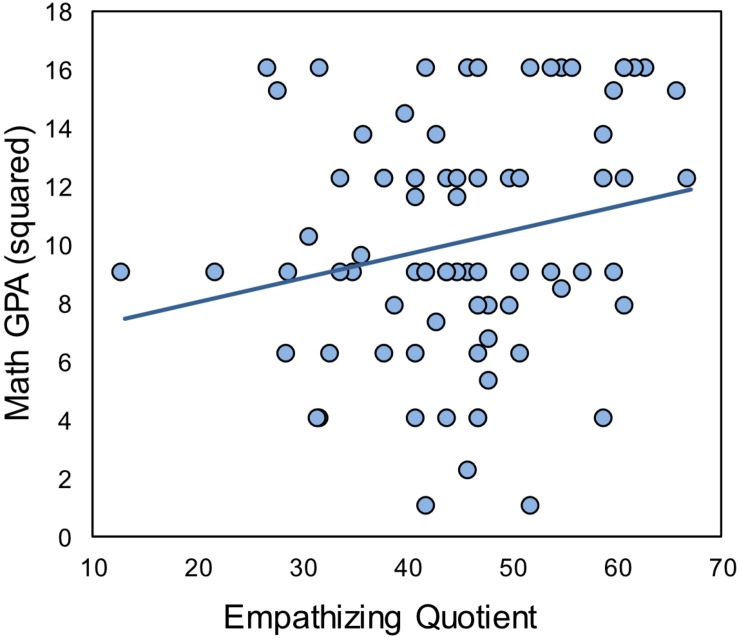
Relations between EQ and Math GPA (Study 2). Participants’ self-report Math GPA (Grade Point Average) squared was marginally correlated with the scores on the Empathy Quotient [*r*(80) = 0.210, *p* = 0.061].

Participant characteristics are listed Table 6. Math achievement, as measured by self-report of Math GPA, was positively related to empathizing in this female-only sample, although the relationship was only marginally significant (Table 7 and Figure 3). This result extends the findings of Study 1 by replicating the positive relationship between EQ and math achievement in women, now in the domain of self-reported math performance at the college-level. Despite the strong positive correlation between EQ and SQ, there was no relationship between SQ and math in this sample. Next, we examined EQ component scores and their relations with Math GPA. Consistent with Study 1, EQ-EE had the strongest relationship of the EQ components with math skills, although it was only marginally significant [*r*(80) = 0.218, *p* = 0.052]. There was no relationship between Math GPA and EQ-CE [*r*(80) = 0.163, *p* = 0.148], or EQ-SS [*r*(80) = 0.086, *p* = 0.451].

**TABLE 7:** Pearson correlations between Math Grade Point Average (GPA), EQ, SQ, and Math Anxiety in women (Study 2).

**Measures**	**EQ**	**SQ**	**Math Anxiety**
Math GPA^1^	0.210	0.136	−0.423^∗∗∗^
EQ		0.475^∗∗∗^	−0.019
SQ			0.130

*Abbreviations and significance as in Table 2. ^1^Only 80 participants reported Math GPA, so correlations with this measure are for *n* = 80. The square of Math GPA was used for correlational analyses. All other measures have *n* = 121. ^∗∗∗^Correlation is significant at the 0.001 level (two-tailed).*

#### Relations Between Empathizing and Math Achievement After Controlling for Math Anxiety

Math anxiety negatively correlated with math achievement, as measured by Math GPA (Table 7), while there was no relationship between math anxiety and EQ, either in the full sample (Table 7), or among the subset of individuals who reported their Math GPA [*r*(80) = −0.114, *p* = 0.315]. Using partial correlations, after controlling for math anxiety, there was no longer even a marginal relationship between EQ and math achievement [*r*(80) = 0.180, *p* = 0.112].

#### Relations Between Attitudes Toward Math, Math Achievement and Empathizing

**FIGURE 4:**
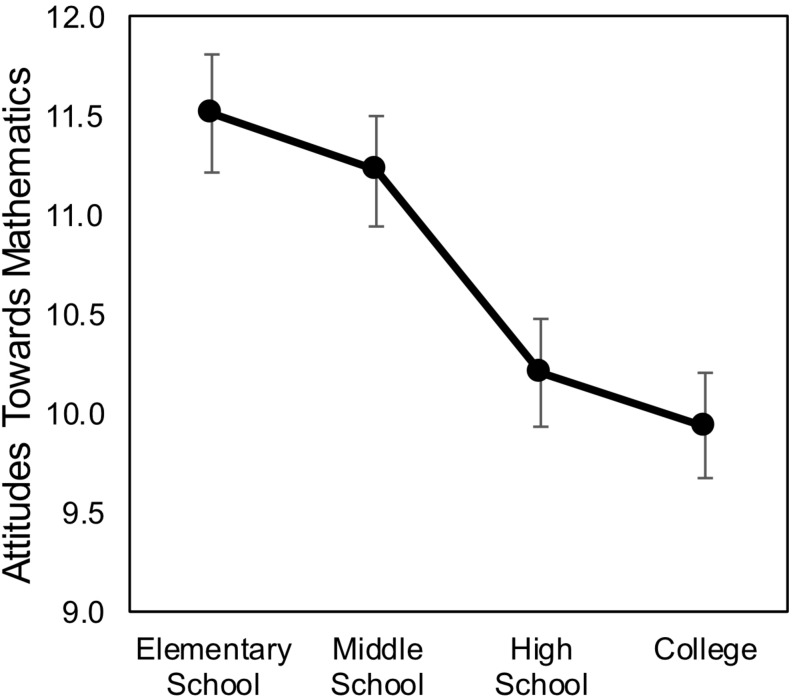
Retrospective attitudes toward math (Study 2). Participants’ attitudes toward mathematics declined over the course of their schooling.

Next, we considered the progression of participants’ attitudes toward mathematics over the course of formal schooling. Participants’ scores on the R-ATM measure declined from elementary, to middle, and into secondary school, and university (Figure 4). A repeated measures ANOVA revealed a significant main effect of time [*F*(1,119) = 17.538, *p* < 0.001]. Within-participant contrasts revealed a marginal decline from elementary to middle school [*F*(1,119) = 3.498, *p* = 0.064], a significant decline from middle to high school [*F*(1,119) = 18.832, *p* < 0.001], but no significant decrease from high school to university [*F*(1,119) = 1.196, *p* = 0.276] (Figure 3).

Next, we examined whether change in participants attitudes toward math was related to math achievement and the relationship between math achievement and EQ. Slope R-ATM was highly correlated with math skills [*r*(80) = 0.425, *p* < 0.001], such that greater declines in attitudes were associated with worse Math GPA. There was also a marginal correlation between EQ and Slope R-ATM [*r*(121) = 0.167, *p* = 0.067], such that participants with higher EQ scores declined less than those with lower EQ scores. Further, partial correlations revealed that controlling for Slope R-ATM, EQ was no longer even marginally related to math skills [*r*(80) = 0.157, *p* = 0.168].

Finally, to quantify the relationship between changing attitudes toward math and EQ, we noted that 27 out of 121 participants had positive Slope R-ATM values, indicating increasing attitudes toward math (and 15 were flat). As would be expected by the correlational analysis, these participants had significantly higher EQ scores than the 79 students who declined [*M* = 49.05, *SD* = 10.89 vs. *M* = 43.64, *SD* = 9.44, *t*(104) = 2.468, *p* = 0.015]. We report this result to illustrate that this difference of 6 points represents more than half of a standard deviation in the distribution of EQ scores (*M* = 44.98, *SD* = 10.40, Table 6). Together these results suggest that individuals with higher EQ scores tend to decline less in their attitudes toward mathematics over the course of schooling.

### Discussion

Consistent with Study 1, we found that math achievement, as measured by self-report of GPA, was positively related to empathizing in a large sample of female college students, although the effect was only marginally significant. Moreover, attitudes toward mathematics declined over the course of schooling, but empathizing acted as a protective factor, with higher EQ levels amongst those whose attitudes improved relative to those who declined. Together these results confirm the unexpected finding of Study 1 and further suggest a mechanism for reconciling these results with those of Escovar et al. (2016), as discussed in the Section “General Discussion.”

## General Discussion

Consistent with prior literature, we found that men had a greater tendency to systemize than women (Groen et al., 2015), however, we did not find a significant difference between the genders in the tendency to empathize. In a recent meta-analysis, Groen et al. (2015) report that effect sizes for the SQ tend to be larger than for EQ, consistent with our results. Interestingly, when we examined the EQ components, women scored significantly higher than men on the emotional empathy component, but not on the cognitive empathy or social skills components (Figure 2), in line with prior work showing that EQ-EE scores display the strongest gender differences (Groen et al., 2015, 2018) among neurotypical individuals.

In two samples, we found that for women, scores on the EQ were positively related to math achievement. In men, correlations were either negative or flat between EQ and math achievement. This result in women is surprising given the finding that higher EQ is related to lower math performance in children, especially girls (Escovar et al., 2016). To better understand the individual differences driving these effects we examined several correlates and components of empathy, including cognitive empathy, emotional empathy, and social skills. We also considered other known predictors of math skills, namely math anxiety and attitudes toward mathematics.

### Social Skills and the Relationship Between Empathizing and Math Achievement, in Women and Men

Empathy and social skills are intimately linked, as the capacities to identify the emotions of others and respond appropriately are crucial precursors to fluent social interactions. While strong social skills could be an asset in a STEM career, prior research suggests that math intensive majors (mathematics, computer science, and physics) have some of the poorest social skill levels among science majors (Baron-Cohen et al., 2001) as do math-intensive majors relative to humanities and social sciences (Valla et al., 2010). Specifically, social skills, as measured by the social interaction factor of the autism quotient (AQ) and the reading the mind’s eye task predicted men’s choice of field, while there was no relationship in women (Valla et al., 2010).

In the current study, we found some support for the negative relationship of empathizing and social skills with math achievement in men (Table 2). However, rather than no relationship, among women we found that better social skills were related to better math performance. In Study 1, we found a significant main effect of SRS scores and significant interaction of gender with SRS in predicting Calculation scores, indicating both that men with worse social skills had better math performance and that women with better social skills had better math performance (Table 5, Model 6). Moreover, SRS explained the interaction of gender and EQ in predicting math skills (Table 5, Model 7). While the EQ component results were less definitive, we did find a marginal positive relationship with Math Fluency in the full group and in women, and a marginal negative relationship with Calculation in men, resulting in a marginal interaction between EQ-SS and gender in predicting Calculation.

Individuals with strong math and verbal skills tend to choose STEM careers at lower rates than individuals with strong math, but moderate verbal skills. This first group is overwhelmingly female and values interacting with people as part of their career (Wang et al., 2013) In contrast, men are over represented among those with strong math but moderate verbal skills, and may pursue STEM majors for lack of other options. Potentially, women with high verbal and math skills may also have strong social skills, further alienating them from less welcoming STEM fields (Ceci et al., 2009). In the current study, we only examined math performance. Further work is needed to assess how social skills might impact not just math achievement but the choice of STEM career.

### Cognitive Empathy and Math Achievement

Cognitive empathy has been used synonymously with theory of mind (Lawrence et al., 2004), which itself has been linked to executive functions (Perner and Lang, 1999), which in turn, strongly predicts math achievement (Clark et al., 2010; Cragg and Gilmore, 2014). Thus, we might expect that the cognitive empathy aspects of empathy would be most closely aligned with math skills. Yet, there were no differences between the genders in scores on the EQ-CE, nor did it correlate with math performance in either sample of women (although there was a marginal interaction between EQ-CE and gender in predicting Calculation scores). Another way to assess the impact of executive functions on the relationship between EQ and math achievement comes from differences between the two standardized math measures. Calculation likely draws more on working memory, while Math Fluency engages task switching (between operations) and inhibitory control (inhibiting answers from different operations). Yet, we did not find consistently stronger relationships for one measure or the other. Future work, targeted at comprehensive cognitive assessments of executive function (and intelligence and other academic skills) is needed to fully characterize the extent, and specificity, of the relationship between EQ and math achievement in women.

### Changing Attitudes Toward Math and Emotional Empathy

The current studies revealed a consistent finding that EQ is positively related to math achievement in college-attending women, yet Escovar et al. (2016) reported a negative relation in children. Clearly, longitudinal studies, which track students’ math achievement and tendency to empathize from elementary school to college are needed to confirm whether, within the same individuals, empathy can shift from a negative to a positive predictor of math skills.

If these results are borne out in such studies, one possible explanation is that empathy itself changes over development, accounting for its changing relationship to math achievement. Decety and Lamm (2006) have characterized empathy as involving both a bottom–up component of sharing the emotions of others, and a top–down component of regulating those emotions. Moreover, while the bottom–up component is developed by the age of 3 years, the top–down component continues to develop from childhood into adolescence and adulthood (Decety, 2010). From this perspective, we would expect that girls with higher empathy might more readily absorb messages about gender stereotypes, impacting math performance in childhood (Beilock et al., 2010). But in adulthood, individuals with high empathy may also have a stronger capacity to regulate emotions and hence distance themselves from these stereotypes. That is, women with higher empathy may be better able to recognize that cultural stereotypes are just rules of society rather than laws of nature, while those with lower empathy may be more likely to internalize these stereotypes.

Two sets of results in the current studies support this conclusion. First, contrary to the general pattern of declining attitudes toward math (Watt, 2004), a subset of women in Study 2 actually increased their positive attitudes toward math, and these had higher EQ scores relative to those who declined. While we cannot assess the direction of this effect, it is possible that women with a greater tendency to empathize may be more able to maintain a positive attitude toward mathematics in the face of negative societal messages. Second, emotional empathy had the highest correlation with Math GPA in Study 2 and the interaction of EQ-EE and gender significantly predicted both math measures in Study 1. These results suggest that the ability to react appropriately to the emotions of others may be a particularly useful skill in navigating these waters.

### Math Anxiety and Math Achievement

Math anxiety is one of the most robust, non-cognitive predictors of math achievement (Ma, 1999). Consistent with this body of research, we found that a self-report measure of math anxiety correlated with math performance, both with standardized measures of math achievement (Study 1) and performance in college-level course work (Study 2). There were no differences between men and women in the rates of math anxiety, nor was the relationship between math anxiety and math achievement modulated by gender, as both genders displayed a negative relationship between math anxiety and math skills.

This pattern of consistent relations between the genders contrasts the finding of no main effect of EQ on math achievement, but instead an interaction between gender and EQ. Further, statistically accounting for anxiety did not change the interaction between EQ and gender in the prediction of Calculation performance in Study 1. However, the inclusion of math anxiety did affect the relationship between math GPA and EQ in Study 2. The negative relationship between math anxiety and math achievement in women was much stronger in Study 2 than in Study 1 (Tables 2, 7), potentially reflecting a greater influence of anxiety on math performance during actual coursework relative to the lower stakes of volunteer laboratory experiment. Larger samples are needed to further asses the unique contributions of EQ on math achievement, independent of the effects of math anxiety. That said, these results further highlight the gendered nature of the relationship between empathizing and math skills, suggesting that empathy may be a particularly important factor for understanding women’s achievement in mathematics.

### Systemizing and Math Achievement

Although the primary focus of these studies was empathizing, we also examined the role of systemizing. In fact, there is considerable theoretical support for the role of systemizing in math achievement, as mathematics is a paradigmatic example of a system. Yet, there is surprisingly little empirical support for the role of systemizing and math achievement. For example, Escovar et al. (2016) found a marginally significant relationship between SQ and performance on the Applied Problems subtest of the WJ-III, which was not significant in either gender. In Study 1, we found some hints of a positive relationship between SQ and math in women (e.g., Math Fluency), but the opposite pattern in men. Moreover, directly comparing these effects found no significant main effects or interactions between gender and SQ in predicting math achievement. In Study 2 there was no relationship between self-report of math achievement and SQ. Together these results suggest that there may be a small effect of SQ on math achievement among women, and studies with larger samples will be needed to detect these relationships.

These findings, coupled with other null results between SQ and math achievement (Morsanyi et al., 2012), raise the possibility that the Systemizing Quotient may be a poor measure of the construct of systemizing. Notably, our findings of strong positive correlations between EQ and SQ, in both men and women, conflicts with both Empathizing – Systemizing Theory (Baron-Cohen, 2009), which suggests they should be negatively correlated, and the Extreme Male Brain Theory (Baron-Cohen, 2002), which suggests they should be independent. In the current study, we used multiple regression to account for any collinearity between the measures. In the broader context, other measures of systemizing might be more informative. For example, Valla and colleagues found that among women, higher scores on the details/pattern factor of the AQ was related to pursuing degrees in fields which involve more systemizing. However, other putative measures of systemizing, such as the embedded figures test and a Go/No Go task were unrelated to field of study in either gender (Valla et al., 2010). How best to conceptualize and measure systemizing remains an open question, especially in applications beyond the original purpose of characterizing autism phenotypes.

## Conclusion

In this study, we examined the relationships between math achievement, and two individual traits, empathizing and systemizing. Contrary to our expectations, we found a positive relationship between math achievement and empathizing in women, that is, higher empathy was related to better math performance, both on standardized measures of math achievement and college-level math attainment. In contrast, among men, lower empathy was related to better math performance. These differential patterns illustrate the complex role gender plays in the path to a STEM career. More broadly, these results contradict the view that traditionally female traits are incompatible with success in mathematics. In fact, rather than empathy acting as an obstacle, it seems to support women’s mathematics achievement, potentially facilitating their pursuit of STEM careers.

## Ethics Statement

This study was carried out in accordance with the recommendations for written (pen/paper or electronic) informed consent of the Arts and Sciences Institutional Review Board, at Rutgers University. The protocol was approved by the Arts and Sciences Institutional Review Board at Rutgers University. All subjects gave written informed consent in accordance with the Declaration of Helsinki.

## Author Contributions

MR-L conceived and designed the studies. NG and ER collected the data for Study 1. NG collected the data for Study 2. MR-L, NG, and ER analyzed the data and wrote the manuscript.

## Conflict of Interest Statement

The authors declare that the research was conducted in the absence of any commercial or financial relationships that could be construed as a potential conflict of interest.

## Footnotes

1 We thank one of the reviewers for suggesting this empathy component analysis.
