# Supplementary material for: Differential Contributions of Empathy to Math Achievement in Women and Men
Source: Front Psychol. 2019 Sep 6;10:1941. doi: 10.3389/fpsyg.2019.01941 (PMC6751398; doi:10.3389/fpsyg.2019.01941)
Supplement: Supplementary file 1 [file Table_1.pdf]

Supplementary Materials

Table S1: Hierarchical regression analysis of Math Skills: Math Fluency. Model 1: Effects of gender, empathy quotient (EQ) and systemizing quotient (SQ). Model 2: additional variance accounted for by interaction of Gender with SQ and EQ.

**Math Fluency**	**B**	**SE**	**Beta**	**t**	**p**
**Model 1** *R^2 = .064*					
Intercept	101.88	1.63		62.64	<.001
Gender	-7.15	2.35	-.259	-3.04	.003
EQ	.19	.12	.14	1.63	.106
SQ	.02	.06	.03	.31	.756
**Model 2** *R^2 = .128, Sig (F-change$_{model1}$) = .035*					
Intercept	102.14	1.64		62.18	<.001
Gender	-7.14	2.31	-.26	-3.09	.002
EQ	.07	.17	.06	.42	.675
SQ	-.09	.09	-.13	-.99	.332
Gender * EQ	.25	.23	.13	1.07	.289
Gender * SQ	.23	.12	.22	1.82	.072

Table S2. Pearson Correlations between Math Achievement and EQ component scores. EQ = Empathy Quotient, CE = Cognitive Empathy, EE = Emotional Empathy, SS = Social Skills

**Women**			
	**EQ-CE**	**EQ-EE**	**EQ-SS**
**Calculation**	-.088	.284*	.124
**Math Fluency**	.028	.306*	.207
**Men**			
	**EQ-CE**	**EQ-EE**	**EQ-SS**
**Calculation**	-.096	-.171	-.228
**Math Fluency**	.065	.063	.096

Table S3. Hierarchical regression analysis of Math Skills with EQ components: Calculation. EQ = Empathy Quotient, CE = Cognitive Empathy, EE = Emotional Empathy, SS = Social Skills

**Calculation**	**B**	**SE**	**Beta**	**t**	**p**
**Model S1** *$R^2 = .057$*					
Intercept	109.05	1.47		74.20	<.001
Gender	-5.31	2.13	-.22	-2.49	.014
EQ-CE	-.39	.27	-.14	-1.45	.149
EQ-EE	.45	.28	.16	1.63	.107
EQ-SS	-.39	.47	-.07	-.84	.400
**Model S2** $R^2 = .139$, *Sig* ($F\text{-change}_{model1}$) $= .007$					
Intercept	108.21	1.467		73.74	<.001
Gender	-5.13	2.06	-.21	-2.49	.014
EQ-CE	-.05	.36	-.02	-.13	.895
EQ-EE	-.31	.40	-.11	-.78	.436
EQ-SS	-.89	.58	-.17	-1.54	.126
Gender * EQ-CE	-.91	.53	-.22	-1.72	.088
Gender * EQ-EE	1.44	.54	.37	2.67	.008
Gender * EQ-SS	1.74	.94	.21	1.85	.066

Table S4 Hierarchical regression analysis of Math Skills with EQ components: Calculation. EQ = Empathy Quotient, CE = Cognitive Empathy, EE = Emotional Empathy, SS = Social Skills

**Math Fluency**	**B**	**SE**	**Beta**	**t**	**p**
**Model S3** *R^2 = .088*					
Intercept	102.03	1.64		62.31	<.001
Gender	-7.43	2.37	-.27	-3.17	.002
EQ-CE	-.11	.30	-.03	-.35	.726
EQ-EE	.36	.31	.11	1.16	.248
EQ-SS	-.71	.52	.12	1.36	.176
**Model S4** *R^2 = .138, Sig ($F\text{-change}_{model1}$) = .056*					
Intercept	101.05	1.66		60.75	<.001
Gender	-7.26	2.34	-.26	-3.10	.002
EQ-CE	.29	.41	.09	.72	.472
EQ-EE	-.48	.45	-.15	-1.05	.496
EQ-SS	.64	.65	.11	.98	.329
Gender * EQ-CE	-.95	.60	-.20	-1.58	.117
Gender * EQ-EE	1.55	.61	.35	2.55	.012
Gender * EQ-SS	.65	1.06	.06	.59	.557
